# NADPH oxidase 4 inhibition is a complementary therapeutic strategy for spinal muscular atrophy

**DOI:** 10.3389/fncel.2023.1242828

**Published:** 2023-09-14

**Authors:** Mirella El Khoury, Olivier Biondi, Gaelle Bruneteau, Delphine Sapaly, Sabrina Bendris, Cynthia Bezier, Zoé Clerc, Elias Abi Akar, Laure Weill, Assaad A. Eid, Frédéric Charbonnier

**Affiliations:** ^1^Faculty of Basic and Biomedical Sciences, University Paris Cité & Inserm UMR_S1124, Paris, France; ^2^Department of Anatomy, Cell Biology and Physiological Sciences, Faculty of Medicine and Medical Center, American University of Beirut, Beirut, Lebanon; ^3^Centre de Recherche en Myologie, UMRS974, Association Institut de Myologie, Sorbonne Université, INSERM, Paris, France; ^4^Département de Neurologie, Centre référent SLA, APHP, Hôpital Pitié-Salpêtrière, Paris, France

**Keywords:** Spinal muscular atrophy, neurodegenerative disorders, oxidative stress, NADPH oxidase 4, GKT137831, severe type SMA-like mice, Setanaxib

## Abstract

**Introduction:**

Spinal muscular atrophy (SMA) is a fatal neurodegenerative disorder, characterized by motor neuron (MN) degeneration and severe muscular atrophy and caused by Survival of Motor Neuron (SMN) depletion. Therapies aimed at increasing SMN in patients have proven their efficiency in alleviating SMA symptoms but not for all patients. Thus, combinational therapies are warranted. Here, we investigated the involvement of NADPH oxidase 4 (NOX4) in SMA-induced spinal MN death and if the modulation of Nox4 activity could be beneficial for SMA patients.

**Methods:**

We analysed in the spinal cord of severe type SMA-like mice before and at the disease onset, the level of oxidative stress and Nox4 expression. Then, we tested the effect of Nox4 inhibition by GKT137831/Setanaxib, a drug presently in clinical development, by intrathecal injection on MN survival and motor behaviour. Finally, we tested if GKT137831/Setanaxib could act synergistically with FDA-validated SMN-upregulating treatment (nusinersen).

**Results:**

We show that NOX4 is overexpressed in SMA and its inhibition by GKT137831/Setanaxib protected spinal MN from SMA-induced degeneration. These improvements were associated with a significant increase in lifespan and motor behaviour of the mice. At the molecular level, GKT137831 activated the pro-survival AKT/CREB signaling pathway, leading to an increase in SMN expression in SMA MNs. Most importantly, we found that the *per os* administration of GKT137831 acted synergistically with a FDA-validated SMN-upregulating treatment.

**Conclusion:**

The pharmacological inhibition of NOX4 by GKT137831/Setanaxib is neuroprotector and could represent a complementary therapeutic strategy to fight against SMA.

## 1. Introduction

Spinal muscular atrophy (SMA) is a fatal neurodegenerative disorder with childhood-onset, characterized by motor-neuron (MN) degeneration leading to severe muscular atrophy (Zerres and Rudnik-Schöneborn, 1995). SMA is caused by *Survival of Motor Neuron 1* (*SMN1*) gene mutation or deletion, resulting in the depletion in SMN protein expression (Lefebvre et al., 1995). In human, *SMN1* gene disruption is partially compensated by the expression of *SMN2*, an *SMN1* gene copy, which produces low amounts of SMN protein due to a polymorphism in the exon7 that prevents its inclusion in 90% of mature transcripts. Exon7-depleted transcripts encode for a truncated form of the SMN protein that is rapidly degraded (Lorson et al., 1999). Thus, the variability in disease severity is mainly linked to the number of *SMN2* gene copies in patients and to the level of their expression (Lorson and Androphy, 2000). To date, the main therapeutic approach for treating SMA consists of increasing full-length SMN expression in SMA patients. Accordingly, three FDA approved therapies are used for treating SMA patients, based either on antisense oligonucleotides (ASOs; Nusinersen/Spinraza) or small molecules (Evrysdi/Risdiplam) that improve exon7 splicing of *SMN2*-derived transcripts (Finkel et al., 2016; Mercuri et al., 2018; Ratni et al., 2018), or on *SMN1* gene replacement using AAV9 (Zolgensma/Onasmnogene abeparvovec; Mendell et al., 2017). Although these treatments improve many SMA symptoms, they do not offer a cure and are associated with some limitations in the therapeutic process. Indeed, patient’s responses to Nusinersen/Spinraza varied between 30 to 70% depending on the clinical trial, and ambulatory patients did not show any improvement with the treatment (Finkel et al., 2017; Mercuri et al., 2018; Wadman et al., 2019; Konersman et al., 2021). Moreover, the use of AAV9 for *SMN1* gene replacement can elicit an immune response and limit the therapeutic benefit by the development of antibodies that target the capsid (Sands, 2011). Furthermore, hepatotoxicity has been reported following intravenous administration of AAV9 (Sands, 2011). Finally, a number of unsuitable off-targets were detected with Evrysdi, leading to perturbations in the transcriptome of targeted tissues (Naryshkin et al., 2014). It remains thus of paramount importance to expand our knowledge in SMA physiopathology as a way to be able to increase the number of treatment options for all patients, including original combination therapies.

One aspect that has retained low attention to date is the potential role of oxidative stress in SMA-induced MN death. Yet, the role of oxidative stress was thoroughly highlighted in several neurodegenerative disorders, such as Parkinson and Alzheimer diseases, and also in another MN-related disease, Amyotrophic Lateral Sclerosis (ALS; Pedersen et al., 1998; Selley, 2002; Barnham et al., 2004). In SMA, the presence of oxidative stress markers has been described in patient’s spinal post-mortem samples (Hayashi et al., 2002; Araki et al., 2003). In addition, higher ROS (Reactive Oxygen Species) levels have been recorded in SMA mouse MNs in relation with defective mitochondrial respiratory chain function (Thelen et al., 2020). However, no oxidative stress could be recorded in SMA iPSC-derived MNs or astrocytes (Patitucci and Ebert, 2016), suggesting that ROS detected in SMA human and mouse spinal samples may be a consequence and not a cause of MN death. In this highly debated context, unraveling mechanisms potentially involved in SMA-induced ROS production may help in reconciling all these data and may open new therapeutical options to improve SMA patient care. Among the different mechanisms that produce ROS in eukaryotic cells, NADPH oxidases (NOXs) have been proposed as major contributors in neurodegeneration (Hernandes and Britto, 2012). Indeed, the NOX family, which include seven members, i.e., NOX1, NOX2, NOX3, NOX4, NOX5, DUOX1, and DUOX2 (Bedard and Krause, 2007), are transmembrane proteins whose only function is to transport electrons across biological membranes by reducing oxygen to superoxide (Babior, 2002; Quinn and Gauss, 2004) or hydrogen peroxide in the case of NOX4 (Serrander et al., 2007). Beyond their role in maintaining intracellular redox status, ROS generated by NOX activity are involved in many cell basic functions, notably the regulation of major intracellular signaling pathways that have been shown altered in SMA, such as AKT and ERK (Branchu et al., 2013; Cavaliere et al., 2013). Unfortunately, no data on the potential involvement of NOXs in SMA physiopathology is currently available. Among the different NOXs, NOX4 has the particularity to be constitutively active, rending the control of its expression particularly critical to maintain ROS homeostasis in NOX4-expressing cells (Martyn et al., 2006; Nisimoto et al., 2010). Moreover, NOX4-produced ROS have been reported as efficient modulators of AKT activity that in turn impacts protein synthesis via the AKT/mTOR pathway (le Belle et al., 2011; Piras et al., 2017). In this signaling cascade, AKT activates the mTOR complex 1 (mTORC1), which in turn inhibits eukaryotic translation initiation factor 4E-binding protein (4E-BP1) and activates ribosomal protein S6 kinase 1 (S6K1), both of which stimulate protein synthesis (Jossé et al., 2016). Noteworthy, translation defects are considered today as an important contributor of SMA physiopathology (Gabanella et al., 2007; Lauria et al., 2020).

In the present study, we investigated the potential involvement of NOX4 in SMA-induced spinal MN degeneration using severe type SMA-like mice at different time-points of the disease. We found that NOX4 was overexpressed in SMA mouse spinal cord and its inhibition by GKT137831/Setanaxib, a drug presently in clinical development (NCT04327089 and NCT05014672), resulted in a significant neuroprotection at the level of the MNs, likely through the activation of the pro-survival AKT/mTOR pathway. Importantly, the GKT137831/Senataxib treatment proved to act synergically with Nusinersen-like ASOs *in vivo*, suggesting that the pharmacological inhibition of NOX4 could represent a complementary therapeutic strategy to SMN gene-based therapies in SMA.

## 2. Methods

### 2.1. Mice and treatment

The knockout transgenic severe type SMA-like mice (FVB/NRj-*SMN*^Δ7/Δ7^, *huSMN2*^+/−^) derived from mice obtained from the Institute of Molecular Biology (Academia Sinica, Taipei, Taiwan). The control mice were heterozygous knocked out for *SMN* and harboring the human *SMN2* transgene (*SMN*^+/Δ7^, *huSMN2*^+/−^; Hsieh-Li et al., 2000). Mouse genotyping was performed using DNA extracted from tail biopsies. Females and males have been equally included in the study. The experiments were performed blinded for genotype, treatment, and molecular and cellular analyses. All experimental mouse groups were randomly constituted, regardless of their weight to minimize bias. A group of severe SMA-like mice was injected intrathecally from post-natal day 1, three times per week, with 4 mg/kg of GKT137831 (Cayman, Ann Arbor, United States) in 0.5 μl/g of methylcellulose and compared with control and severe type SMA-like mice injected from post-natal day 1 with 0.5 μl/g of methylcellulose. A group of severe SMA-like mice was treated by a single ICV injection of 2 μg of ASO-10-27 (ISIS-SMNRx; Eurogentec) as described by Rigo et al. (2014) at day 1 combined with a repeated *per os* administration three times a week of 40 mg/kg GKT137831 in 0.5 μl/g methylcellulose or of 0.5 μl/g methylcellulose. For the cellular and molecular studies, mice were euthanized and dissected at post-natal day 8. All the experiments were performed in the faculty animal facility (BioMedTech Facilities, CNRS UMS 2009, INSERM US 36). The care and treatment of animals followed the national authority (French Ministry of Research and Technology) guidelines for the detention, use, and ethical treatment of laboratory animals.

### 2.2. Animal behavior analysis

Grip strength was performed in the forelimb of vehicle- and GKT 137831-treated severe type SMA-like mice from post-natal day 5 to death. The time spent holding onto a thin metal rod suspended in mid-air was measured. Each mouse was subjected to five successive attempts separated by a 30 s rest period. Only the maximal value was recorded.

The ambulatory behavior was assessed in an open-field test for all groups. The equipment consisted of a box measuring 15 × 15 cm for mice aging 5 and 6 days and 28 × 28 × 5 cm for mice aged 7 days and older. The floor of the arena was divided, respectively, into 25 and 16 equal squares. The squares adjacent to the walls were referred to as periphery, and the four remaining squares were referred to as center. The mice were tested individually. Each mouse was initially placed in the center of the open field and allowed to move freely for 5 min with repetitive tail pinching every 15 s. The number of peripheral and central square crossings were recorded manually.

### 2.3. ROS measurement with DHE

Freshly dissected tissues were place in OCT and frozen in cooled Isopentane solution. Tissues were sectioned to a thickness of 14 μm, placed on glass slides, and kept frozen. After quick thaw, tissues were immediately washed with PBS and incubated with 15 μm of Dihydroethidium (DHE, Invitrogen D11347) diluted in PBS for 30 min at 37°C in humid dark container. Slides were rinsed in PBS and mounted with Fluoromount-G (Invitrogen) supplemented with Bis-Benzimide (1:1000, Sigma B2261). Intensity measurements were performed with image J.

### 2.4. NADPH oxidase activity

Proteins were extracted from the lumbar spinal cord using the electrical potter in lysis buffer [20 mM KH2PO4 (pH 7.0), 1 mM EGTA, 1 mM Phenylmethylsulfonyl fluoride, 10 μg/ml Aprotinin, and 0.5 μg/ml Leupeptin]. Protein content was measured using the Bio-Rad protein assay reagent and then25 μg of proteins were added to 50 mM phosphate buffer (pH 7.0) containing 1 mM EGTA, 150 mM sucrose, 5 μm lucigenin (behaving as the electron acceptor), and 100 μm NADPH (acting as the substrate for the NADPH oxidase). Photon emission expressed as relative light units (RLU) was measured every 30 s for 8 repetitions using Enspire (PerkinElmer). Superoxide production was expressed as relative light units/min/mg of protein.

### 2.5. Protein extraction

Lumbar spinal cords were dissected and immediately frozen in liquid nitrogen. Lumbar spinal cords were homogenized using metal beads with a TissueLyser apparatus in RIPA buffer (50 mM Tris–HCL, 150 mM NACL, 0.1% Sodium Dodecyl Sulfate, 1% Nonidet P-40, 0.5% Sodium Deoxycholate) supplemented with protease and phosphatase inhibitors. Homogenates were then centrifuged at 17,000 rcf for 30 min at 4°C. Protein concentration of the clarified homogenate was determined on all samples using the Lowry method (Lowry et al., 1951).

### 2.6. Western blot analysis

30 μg of whole cell extract samples were fractionated either by 10% (NOX4 and NOX1) or 4–15% (pAKT, AKT, pERK 1/2, ERK 1/2, pCREB, CREB, p62, p4EBP1, pS6) SDS-PAGE electrophoresis. The separated proteins were transferred on PVDF or nitrocellulose membranes (Bio-Rad Laboratories). Membranes were blocked with 5% BSA for phosphorylated proteins, or skimmed milk for the remaining proteins. Each of the following primary antibodies, including monoclonal mouse anti-SMN (1:5000; BD Biosciences 610,646), rabbit monoclonal Anti-NOX4 (1:1000, Abcam 133,303), rabbit polyclonal anti-NOX1 (1:1000, Santa Cruz 25,545) rabbit polyclonal anti-phospho-AKT (Ser 473; 1:1000, cell signaling 9271S), rabbit polyclonal anti-AKT (1:1000, cell signaling 9272S), rabbit monoclonal anti-phospho-ERK1/2 (Thr202/Tyr204, Thr185/Tyr187; 1:500, Millipore 05-797R), rabbit polyclonal Anti-MAPK ½ (ERK1/ERK2; 1:500, Sigma ABS44), rabbit polyclonal anti-phospho-CREB (Ser133; 1:500, Millipore 06–519), mouse monoclonal anti-Nucleoporin p62 (1:1000, BD Biosciences 610,497), rabbit monoclonal anti-phospho-4E-BP1 (Thr37/46; 1:1000, Cell Signaling 2855p), rabbit monoclonal anti-phospho-S6 Ribosomal Protein (Ser240/244; 1:1000, Cell Signaling 5,364),or mouse monoclonal anti-α-TUBULIN antibody (1:20.000, Sigma T6074) was incubated overnight at +4°C in 5% BSA constituted in TBS pH 7.4 supplemented with 0.05% Tween 20. The blots were then incubated with either polyclonal goat anti-mouse antibody (1:10000, Biorad 1,706,516) or polyclonal goat anti-rabbit antibody (1:10000, Jackson Immunoresearch 111–035-003). In some occasions, membranes were stripped with 100 mM b-Mercaptoethanol, 2% SDS, 62.5 mM Tris–HCl, pH 6.7 for 30 min at 55°C with agitation, and then blocked with 5% milk and blotted again with other primary antibodies. Bands were visualized by enhanced chemiluminescence. Intensity analysis was performed using NIH ImageJ software.

### 2.7. Histological and immunofluorescence analysis

Euthanized mice were dissected and harvested tissues were either placed in OCT and frozen in cooled Isopentane solution or incubated overnight in 4% PFA solution then placed in PBS after washing.

For MN counting, the lumbar spinal cords were embedded in 4% Agarose solution in sterilized water and placed at 4°C until solidification. 50 μm sections were then performed using a vibratome on the whole length of the sample. Tissue sections were incubated for 1 h at room temperature in a blocking solution [2% BSA, 5% FBS with 0.5% Triton X-100 and 0.1% Tween-20 in Tris-buffered saline (TBS)]. Motor neuron labeling was performed using a Choline Acetyltransferase (ChAT) primary antibody (polyclonal goat anti-ChAT; 1:400; Millipore Bioscience Research Reagents) for 2 days at 4°C. Sections were washed between each subsequent step with TBS. Sections were subsequently incubated with an Alexa Fluor 568 donkey anti-goat IgG (1:400; Jackson ImmunoResearch) for 1 h at room temperature. The sections were then rinsed and incubated with Bis-Benzimide (1:1000, sigma B2261) in PBS for 3 mins and washed three times for 10 min 0.1% Tween-20 in TBS and mounted with Fluoromount-G (Invitrogen).

For motor endplate labeling, muscle sections were prepared with previously described agarose embedding with a thickness of 75 micrometers. Sections were stained using Alexa Fluor 568-conjugated -Bungarotoxin (4 mg/ml in 0.5% Triton X-100 and 0.1% Tween-20 PBS with 4% bovine serum albumin, and 5% donkey serum). Presynaptic motor nerve terminals were stained with isoform of Neurofilament light protein (monoclonal rabbit 1:500; Millipore Bioscience Research Reagents) and Snap25 (1:200; Bio-Techne) after 4 h of blocking. The whole-mount preparations were subsequently incubated with an Alexa Fluor 488 goat anti-rabbit IgG (1:400; Invitrogen) for 1 h at room temperature then with Bis-Benzimide (1:1000, sigma B2261) in PBS for 3 mins.

For SMN, NRF2, and NOX4 immunodetection, frozen sections of 14 μm thickness were thawed, fixed for 20 min with 4% paraformaldehyde saturated, and permeabilized with 2% BSA, 5% donkey serum in 0.5% Triton X-100 and 0.1% Tween-20 TBS (For SMN, permeabilization media consist of TBS with 1% Tween-20 and Triton X-100). Then, antibodies were incubated [1:400, rabbit anti-SMN #502 (Sapaly et al., 2018); rabbit anti-NRF2 Novus Biological NBP1-32822; rabbit anti NOX4, Abcam 133,303] in blocking solution for 48 h. Then, the sections were incubated with an Alexa Fluor 488 donkey anti-rabbit IgG. Sections were then incubated with Bis-Benzimide (1:1000, sigma B2261) in PBS for 1 min, washed and mounted with Fluoromount-G (Invitrogen).

All counts were performed using NIH ImageJ software and identical brightness, contrast, and color balance adjustments were applied to all groups.

### 2.8. Microscopy

All immunofluorescence images were collected with a CMOS camera (ORCA Flash 2.8, Hamamatsu Photonics France, Massy, France) mounted on a Zeiss AxioObserver microscope (Z1, Carl Zeiss SAS, Le Pecq, France) using the ZEN 2012 software (Carl Zeiss SAS) with 200 (X20 Zeiss EC-Plan-Apo NA 0.8) magnifications.

### 2.9. Statistical analysis

All data are expressed as means and standard deviation (± SD) of n different mice. For endpoint studies, a non-parametric Mann–Whitney U test were performed (Prism 7, GraphPad Software, Inc). Statistical significance was considered when statistical power exceeds 95% in two-tailed calculation (*p* < 0.05; AnaStats.fr, France).

Survival analysis was performed using log rank Mantel-Cox analysis. Statistical significance was considered when statistical power exceeds 95% in two-tailed calculation (*p* < 0.05).

## 3. Results

### 3.1. NOX4 induces ROS production in the spinal cord of severe type SMA-like mice

**Figure 1:**
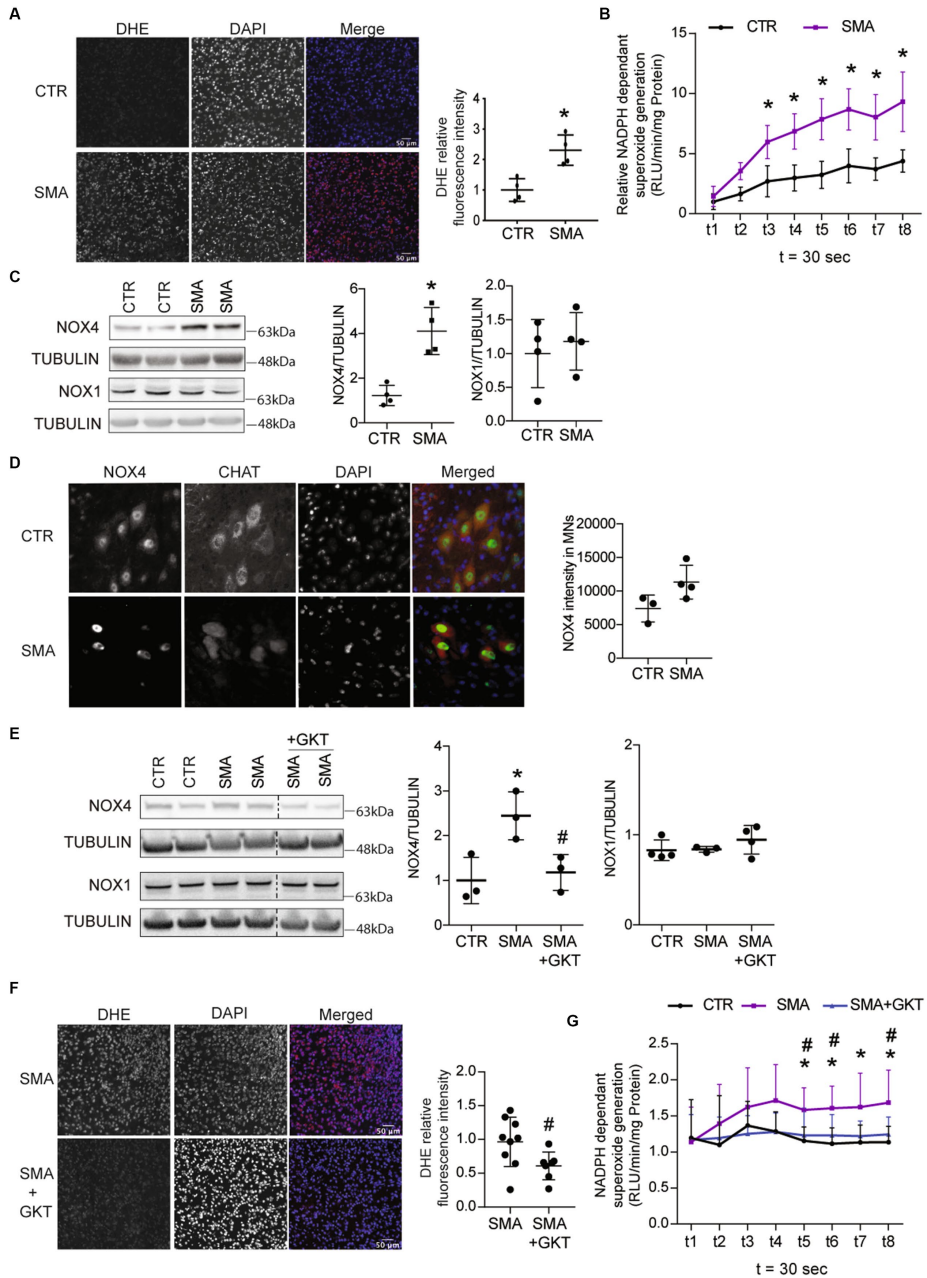
ROS accumulation is associated with NOX4 overexpression in the spinal cord of spinal muscular atrophy (SMA)-like mice. Evaluation of superoxide accumulation by DHE staining (scale bar 50 μm, **A**) and of NADPH-dependent superoxide generation **(B)** in the spinal cord of severe type SMA-like mice compared to control P8 (*n* = 4). Western blot (left panel) and quantification (right panel) of NOX4 and NOX1 protein expressions in the spinal cord of severe type SMA-like mice compared control mice at p8 **(C)**. Immunodetection of Nox4 (Green) in ChAT-positive MNs (Red) in the lumbar spinal cord of severe type SMA-like mice and controls at p8 (scale bar 20 μm; left panel) and quantification of NOX4 intensity in MN (right panel, **D**). Western blot analysis and quantification of NOX4 and NOX1 proteins in the spinal cord of GKT137831- or vehicle-treated severe type SMA-like mice compared to control mice (*n* = 6 and *n* = 4, respectively; **E**). Evaluation of superoxide accumulation by DHE staining (**F**; *n* = 7; scale bar 50 μm) and of NADPH-dependent superoxide generation (**G**; *n* = 5) in the spinal cord of GKT137831- or vehicle-treated severe type SMA-like mice compared to control mice at P8. *p < 0.05 vs. Control and #*p* < 0.05 vs. SMA; error bars indicate standard deviation (SD); Vertical dotted lines indicate that the pictures have been spliced from the same blot.

Autopsies of SMA patients have shown ROS accumulation in the spinal MNs (Hayashi et al., 2002; Araki et al., 2003), but the source of these ROS remains elusive. To address this question, we first measured ROS levels in the spinal cord of severe type SMA-like mice, at P6 (presymptomatic stage) and P8 (disease onset), using Dihydroethidium (DHE) staining and NADPH oxidase activity assessment. We found that ROS started to accumulate at P8 in SMA mouse spinal cord (Figure 1A; Supplementary Figure S1A), thus at the beginning of the disease symptomatic phase in these mice (Hsieh-Li et al., 2000). Interestingly, this accumulation of ROS timely coincided with a significant increase in NADPH oxidase activity (Figure 1B; Supplementary Figure S1B).

Then, we wondered which NOXs could be involved in ROS production and in the increase of NADPH oxidase activity in the spinal cord of severe type SMA-like mice. We found that NOX4 protein levels increased by 4-fold at P8 in severe type SMA-like mice spinal cord compared to controls (Figure 1C), while no difference in NOX4 expression could be recorded at P6 (Supplementary Figure S1C). By contrast, NOX1 displayed no variation (Figure 1C). To evaluate whether NOX4 overexpression could be detected in SMA spinal MNs, we performed a NOX4 immunostaining in Choline-Acetyl-Transferase (ChAT)-stained MNs in the spinal cord of severe type SMA-like mice. As shown in Figure 1D, MNs are NOX4-expressing cells and NOX4 intensity in SMA MNs tends to be higher than in the controls. In order to evaluate the level of NOX4 involvement in ROS production, we analyzed the effects on ROS accumulation of the pharmacological inhibition of NOX4 directly in spinal cord. We selected GKT137831 (Setanaxib), a NOX4/1 inhibitor from the Pyrazolopyridine chemicals series (Aoyama et al., 2012). We treated a population of severe type SMA-like mice three times per week by intrathecal injection with 4 mg.g^−1^ of GKT137831 or vehicle, and analyzed at P8 the ROS production in the spinal cord. First of all, the GKT137831 treatment restored the level of NOX4 protein in severe type SMA-like mice spinal cord when compared to controls (Figure 1E), while no change in NOX1 expression was detected (Figure 1G), suggesting that GKT137831 acts mostly by inhibiting NOX4 activity. Interestingly, we found that the GKT137831 treatment resulted in a significant decrease in ROS levels in the severe type SMA-like mice spinal cord, compared to vehicle-treated mice, as revealed by DHE staining (Figure 1F). This decrease in ROS accumulation was expectedly associated with a significant decrease in NADPH oxidase activity (Figure 1G).

Thus, taken together, these data suggested that NOX4 could be a major source of ROS production in the spinal cord of severe type SMA-like mice. Interestingly, the NOX4/1 inhibitor GKT137831 proved to normalize ROS levels in the severe type SMA-like mice spinal cord, providing an opportunity to further explore the potential role of oxidative stress in SMA MN degeneration.

### 3.2. NOX4 inhibition limits MN loss and neuromuscular junction defects in severe type SMA-like mice

**Figure 2:**
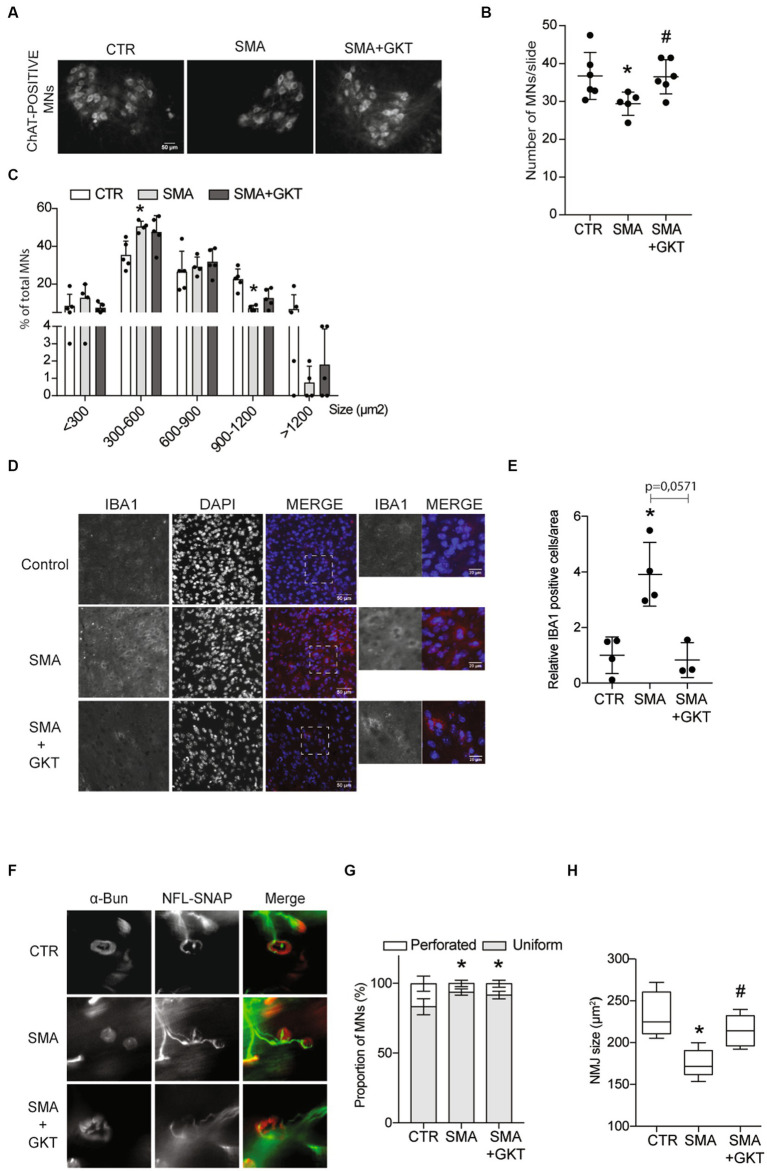
NOX4 inhibition limits MN loss, microgliosis and neuromuscular junction defects in severe type SMA-like mice. Immunodetection of ChAT-positive MNs in the ventral horn of the L1–L6 lumbar spinal cord of GKT137831 or vehicle-treated severe type SMA-like mice at P8, compared to age-matched controls (*n* = 4). Representative images are shown (Scale bar, 50 μm; **A**) and quantitative analysis of the number **(B)** and cell body area **(C)** of MNs per section. Immunofluorescence of IBA1-positive cells in the spinal cord of GKT137831- or vehicle-treated severe type SMA-like mice compared to control mice at P8 (*n* = 4; Scale bar 50 μm; **D**). Zoom panels (Scale bar 20 μm) show the magnified regions that are indicated with the dotted square. Quantification of IBA1 positive cells by area (E)*p < 0.05; error bars indicate SD. Analysis of the neuromuscular junction (NMJ) in the Tibialis of GKT137831- or vehicle-treated severe type SMA-like mice compared to control mice at P8. Immunofluorescent analyses of the NMJs using anti-SNAP25 and anti-neurofilament antibodies in red and labeled alpha-bungarotoxin (BGTX) staining for the acetylcholine receptors (AChRs) in green (Scale bar 20 μm; **F**). Proportion of the NMJ morphology (perforated or uniform; **G**) and quantification of the areas of the AChR clusters (**H**; *n* = 5) *p < 0.05 vs. Control and #*p* < 0.05 vs. SMA; error bars indicate SD.

We then questioned whether GKT137831-mediated ROS inhibition could have an impact on the survival of SMA MNs. Therefore, we assessed the populations of spinal MNs by counting the residual number of ChAT-positive cells in the ventral horn of the L1–L6 lumbar spinal cord of GKT137831 or vehicle-treated severe type SMA-like mice at P8, compared to age-matched controls (Figures 2A,B). Interestingly, while a significant 20% decrease in spinal MN number could be detected in vehicle-treated severe type SMA-like mice at P8, the number of spinal MNs was no longer significantly different from control mice in GKT137831-treated severe type SMA-like mice (Figures 2A,B), suggesting that NOX4 inhibition exerted a significant neuroprotection of SMA MNs. To better characterize the neuroprotective effects of NOX4 inhibition, we analyzed the different spinal MN subpopulations, based on their soma size distribution (Figure 2C). In vehicle-treated severe type SMA-like mice, we found a significant increase in the percentage of intermediate soma-sized SMA MNs (area between 300 and 600 μm^2^), associated with a significant decrease in the percentage of large soma-sized SMA MNs (area greater than 900 μm^2^) in comparison to controls, as previously reported (Biondi et al., 2010). Interestingly, the SMA-induced shift in MN subpopulations toward smaller-sized cells proved to be significantly limited by the GKT137831 treatment, since no more significant difference could be detected in the distribution of MN subpopulations in the spinal cord of GKT137831-treated severe type SMA-like mice compared to controls.

Since microglia are major NOX-dependent ROS-producing cells in the CNS (Brown and Vilalta, 2015) and SMA has been shown to induce microgliosis (Ando et al., 2020), thought to participate in neuron degeneration (Lull and Block, 2010; Hickman et al., 2018), we investigated whether NOX4 inhibition by GKT137831 could limit also the activation of the microglia in the spinal cord of severe type SMA-like mice. Hence, we assessed microglia activation by counting IBA1-positive cells in the lumbar spinal cord of GKT137831- or vehicle-treated severe type SMA-like mice at P8, compared to age-matched controls (Figures 2D,E). A 4-fold increase of IBA1-positive cells could be recorded in vehicle-treated severe type SMA-like mice spinal cord in comparison to controls (Figures 2D,E). Interestingly, the GKT137831 treatment resulted in a significant decrease of the SMA-induced microgliosis, with a number of IBA1-positive cells that reached levels comparable to controls.

In parallel, we studied whether MN protection and microglia limitation induced by the GKT137831 treatment was efficient in limiting neuromuscular junction (NMJ) alterations, which is considered today as a cellular hallmark of SMA physiopathology (Goulet et al., 2013). We analyzed NMJ morphology and size in the *Tibialis* muscle of GKT137831 or vehicle-treated severe type SMA-like mice at P8, compared to age-matched controls (Figure 2F). While the shape of the NMJ was not significantly modified by the GKT137831 treatment and remained less perforated than in controls (Figure 2G), we found that NMJ atrophy proved to be significantly limited by the treatment, and the SMA-induced decrease in NMJ area was no longer significantly different from controls in GKT137831-treated SMA muscles (Figure 2H).

Taken together, these results provide the first lines of evidence that limiting ROS accumulation in SMA spinal cord through NOX4 inhibition is protective for MNs, hinders SMA-induced microgliosis and led for the relative maintenance of NMJs.

### 3.3. NOX4 inhibition promotes activation of AKT and mTOR pathways in the spinal cord of severe type SMA-like mice

**Figure 3:**
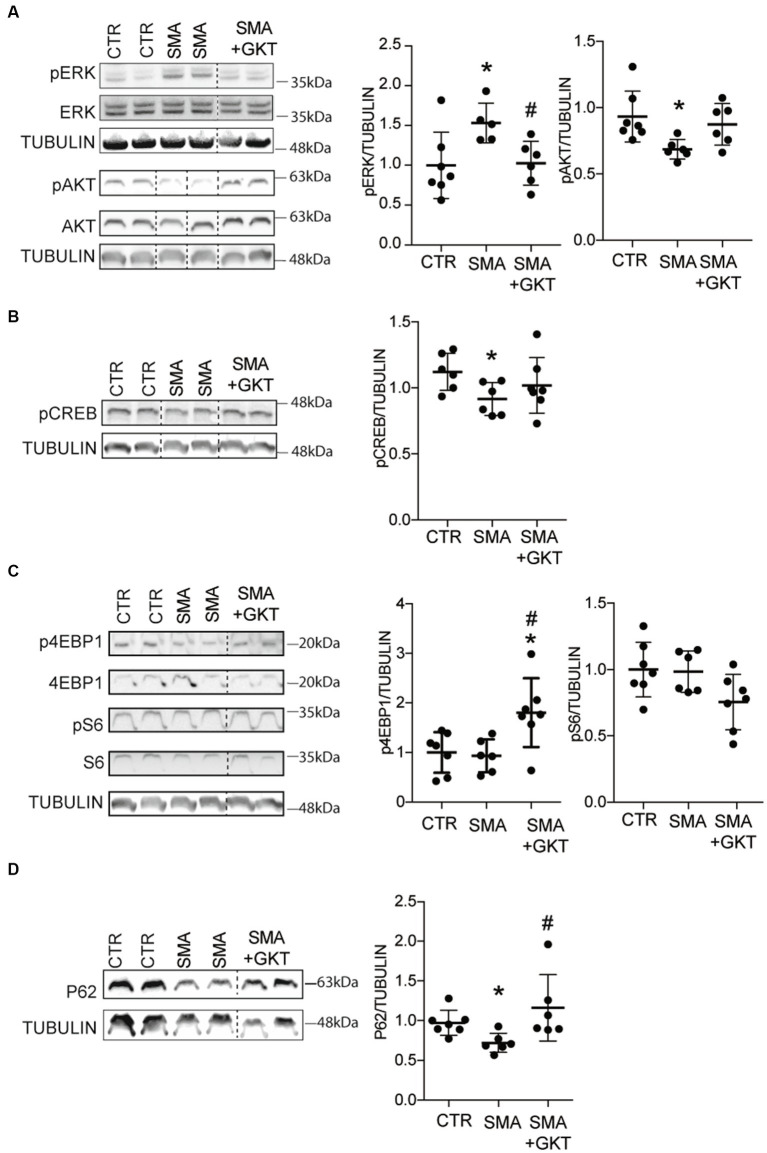
NOX4 inhibition activates AKT and mTOR pathways in the spinal cord of SMA-like mice. Western blot analysis of ERK and AKT **(A)**, CREB **(B)**, p4E-BP1 and S6 **(C)** phosphorylation patterns and of P62 protein expression **(D)** in the ventral horn of the L1–L6 lumbar spinal cord of GKT137831- or vehicle-treated severe type SMA-like mice at P8, compared to age-matched controls (*n* = 6). *p < 0.05 vs. Control and #*p* < 0.05 vs. SMA; Error bars indicate SD.

We next sought to identify the molecular mechanisms underlying the pro-survival effects of the GKT137831 treatment on SMA MNs. NOXs have been previously shown to modulate several master signaling pathways that control MN survival such as ERK or AKT pathways (Cavaliere et al., 2013; Zhao et al., 2015) and their downstream pathways including the signaling platform mTORC1 (le Belle et al., 2011). We first evaluated the phosphorylation profile of ERK and AKT (Figure 3A) in the ventral horn of the L1–L6 lumbar spinal cord of GKT137831 or vehicle-treated severe type SMA-like mice at P8, compared to age-matched controls. We found that the GKT137831 treatment was efficient in rebalancing the activation pattern of the two kinases, activating the SMA-induced down-regulated AKT and inhibiting the SMA-induced over-regulated ERK. Noteworthy, AKT and ERK phosphorylation pattern was no more significantly different from controls in GKT137831-treated SMA mouse spinal cord. Moreover, the activation pattern of the transcription factor CREB, that lays downstream AKT in the spinal cord (Branchu et al., 2013), paralleled the activation of AKT, with a SMA-induced down-regulation counteracted by the GKT137831 treatment (Figure 3B).

Then, we investigated whether the GKT137831-induced activation of AKT could activate in turn the mTORC1 cascade, which is notably involved in protein synthesis through modulation of ribosomal S6 protein kinase 1 (S6K1) that activate S6 proteins, and eukaryotic initiation factor 4E-binding protein 1 (4E-BP1). Thus, we evaluated the phosphorylation profile of 4EBP1 and S6 proteins in the ventral horn of the L1–L6 lumbar spinal cord of GKT137831 or vehicle-treated severe type SMA-like mice at P8, compared to age-matched controls (Figure 3C). While the levels of phosphorylation of 4E-PB1 ans S6 proteins were the same between control and SMA, we found that the GKT137831 treatment promoted the phosphorylation of 4E-PB1 protein, but not of S6 protein, which activation profile remained unchanged compared to vehicle-treated severe type SMA-like mice mouse spinal cord. These results suggest that the 4E-PB1 branch of the mTORC1 pathway is activated following NOX4 inhibition.

We next wondered whether the autophagy process, which is under the control of mTORC1 (Hosokawa et al., 2009), was modulated by the GKT137831 treatment. Thus, we evaluated the expression of p62, a protein involved in phagosome assembly, in the ventral horn of the L1–L6 lumbar spinal cord of GKT137831- or vehicle-treated severe type SMA-like mice at P8, compared to age-matched controls (Figure 3D). We found that the decreased levels of p62 protein expression in the spinal cord of severe type SMA-like mice compared to controls was significantly limited by the GKT137831 treatment. These data suggested that autophagy might be overactivated in SMA mouse spinal cord, as previously described (Sansa et al., 2021) and rebalanced by the GKT137831 treatment.

Finally, since mitochondria alterations led to ROS accumulation in SMA (Thelen et al., 2020), on the one hand, and NOX4 impacted mitochondria biogenesis and bioenergetics (Bernard et al., 2017) on the other, we explored whether the GKT137831 treatment impacted the signaling pathways that control the number and function of mitochondria. NOX4 has been shown to inhibit mitochondria biogenesis and bioenergetics by targeting the Nuclear Factor Erythroid-derived 2 Related Factor 2 (NRF2), a powerful transcriptional activator of the mitochondrial transcription factor A (TFAM), which controls mitochondrial biogenesis (Larsson et al., 1994; Kang et al., 2007; Bernard et al., 2017). Thus, we analyzed the levels of TFAM mRNA expression in the ventral horn of the L1–L6 lumbar spinal cord of GKT137831- or vehicle-treated severe type SMA-like mice at P8, compared to age-matched controls (Supplementary Figure S2A). No significant difference in the expression of TFAM gene could be evidenced between mouse groups. We next evaluated the levels of NRF2 activation by counting the number of NRF2-positive dots in the nucleus of spinal MNs (Supplementary Figures S2B,C). Our results revealed no change in NRF2 translocation in MN nucleus, whatever the conditions studied. Thus, all these results suggest that a modulation of the NRF2-TFAM pathway is unlikely involved in the effects provided by NOX4 inhibition in the spinal cord of severe type SMA-like mice.

Taken altogether, these results suggest that NOX4 inhibition directly in severe type SMA-like mice spinal cord efficiently activates the pro-survival AKT-CREB pathway, concurring to the neuroprotection recorded at the level of the MNs. Moreover, NOX4 inhibition could activate one branch of the mTORC1 pathway involved in protein synthesis, raising interesting questions about the potential impact of the GKT137831 treatment on SMN protein expression.

### 3.4. NOX4 inhibition promoted SMN expression in the spinal cord of severe type SMA-like mice

**Figure 4:**
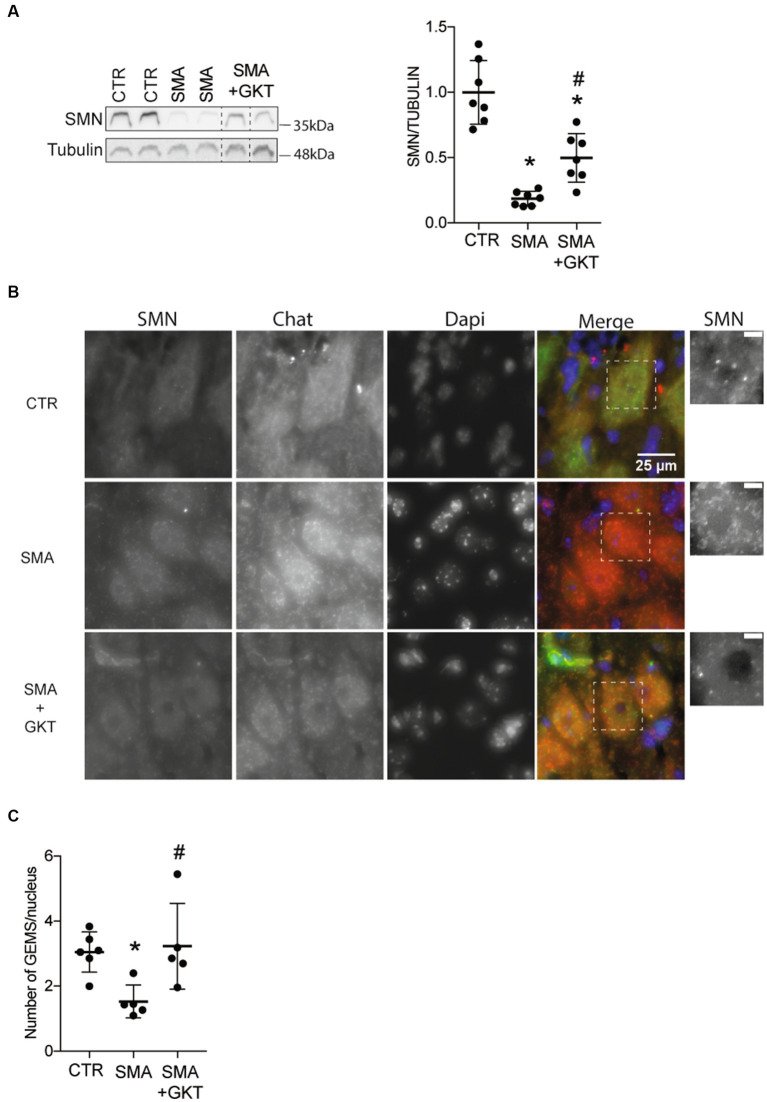
NOX4 inhibition promotes SMN expression in the spinal cord of severe type SMA-like mice. Western blot analysis of SMN protein expression in the ventral horn of the L1–L6 lumbar spinal cord of GKT137831- or vehicle-treated severe type SMA-like mice at P8, compared to age-matched controls (*n* = 7; *p < 0.05; error bars indicates SD; **A**). Immunodetection of SMN in ChAT-positive MNs in the ventral horn of the L1–L6 lumbar spinal cord of GKT137831- or vehicle-treated severe type SMA-like mice at P8, compared to age-matched controls (**B**; Scale bar: 25 μm). Zoom panels (Scale bar: 5 μm) show the magnified regions that are indicated with the dotted square. Number of Gems per nucleus in MN nucleus (*n* = 5; **C**). *p < 0.05 vs. Control and #*p* < 0.05 vs. SMA. Error bars indicate SD.

The evidence of the GKT137831-induced modulation on signaling pathways involved in the regulation of SMN expression prompted us to investigate the levels of SMN protein expression in the ventral horn of the L1–L6 lumbar spinal cord of GKT137831 or vehicle-treated severe type SMA-like mice at P8, compared to age-matched controls (Figure 4). We first analyzed SMN protein expression by western blot, and found that the GKT137831 treatment induced a significant increase in SMN protein expression in severe type SMA-like mice spinal cord compared to vehicle-treated counterparts (Figure 4A). We next investigated whether this effect of GKT137831 in inducing SMN protein expression could be specifically recorded in MNs. We then performed an immunodetection of SMN in ChAT-positive cells and counted the number of Gemini of Coiled bodies (Gems) in the spinal cord of GKT137831 or vehicle-treated severe type SMA-like mice at P8, compared to age-matched controls (Figures 4B,C). Importantly, we found that the GKT137831 treatment was able to induce a significant increase in the number of Gems in SMA MNs, which, with an average of 3 Gems per MN nucleus, reached a level comparable to controls (Figure 4C).

Thus, taken together, these results suggest that SMA-induced NOX4 overexpression in the mouse spinal cord may worsen SMN depletion and, most importantly, that NOX4 inhibition is sufficient to significantly increase SMN expression levels in SMA MNs.

### 3.5. NOX4 inhibition improves the motor behavior and the life span of severe type SMA-like mice

**Figure 5:**
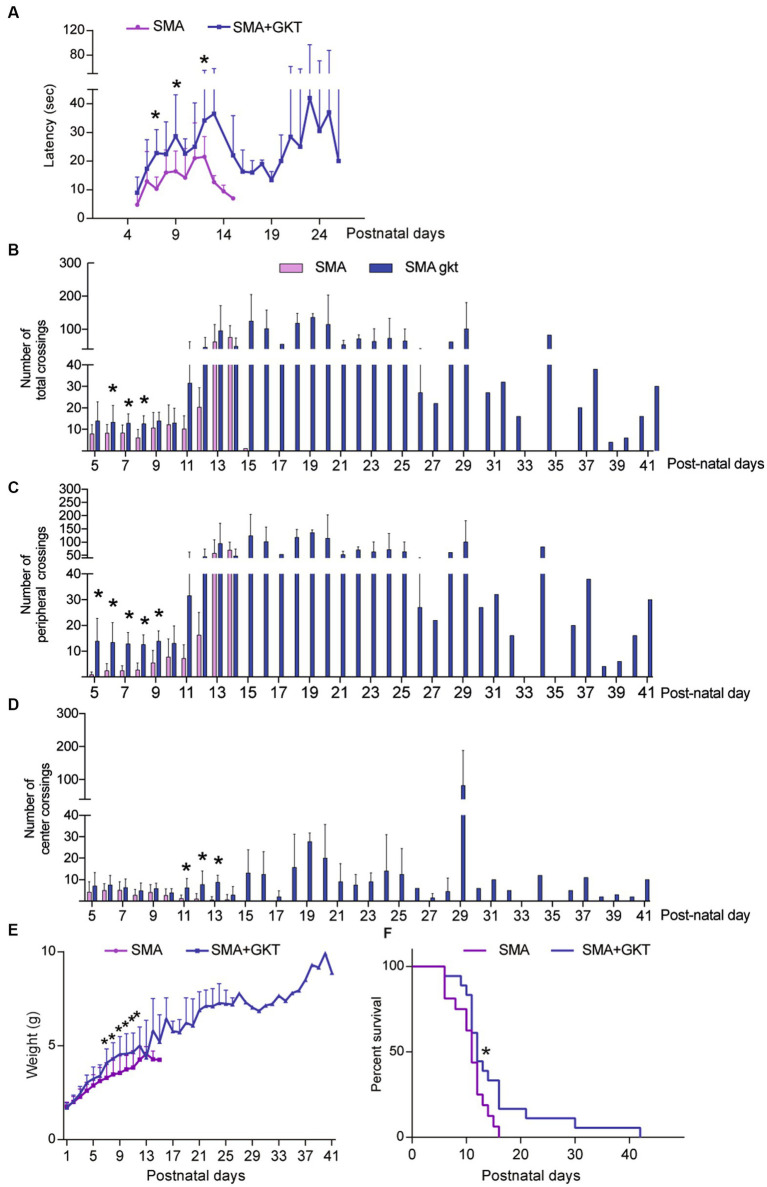
NOX4 inhibition improves motor behavior and life span of severe type SMA-like mice. Grip test **(A)**, number of total **(B)**, peripheral **(C)** and central crossings **(D)** in an open field during 5 min, body weight **(E)**, and life span **(F)** curves after intrathecal injection of GKT137831 (Blue) or vehicle (Pink) of severe type SMA-like mice compared to age-matched controls. For grip test and Open field, *n* = 15 and for body weight and life span (*n* = 19). *p < 0.05 vs. SMA; error bars indicate SD.

We next questioned whether the benefits induced by the GKT137831 intrathecal treatment at the cellular level could improve the motor behavior of severe type SMA-like mice. We first tested the grip latency of the mice during the time course of the disease and found that the GKT137831 treatment significantly increased the grip time of severe type SMA-like mice since the age of P7 (Figure 5A). We next analyzed the mouse spontaneous activity in an open field, that reflect both motor and behavioral phenotypes. We found that the GKT137831 treatment significantly improved exploration activity of severe type SMA-like mice, which progressively increased with age, as evidenced by the total number of crossings that doubled from P6 to P9, the increased exploration in the periphery of the field compared to vehicle-treated SMA-like mice and in the center of the field (Figures 5B–D).

The beneficial effects of the GKT137831 treatment on motor behavior were associated with a significant improvement of the mouse body weight curve, with a significant increase in the body mass since P5, compared to vehicle-treated counterparts (Figure 5C). Interestingly, the GKT137831-treated severe type SMA-like mice maintained their body weight until their death.

Finally, we analyzed the effect of the GKT137831 treatment on the lifespan of severe type SMA-like mice. We found that the median survival was significantly increased between mouse populations (11 and 12 days for vehicle- and GKT137831-treated severe type SMA-like mice, respectively) and the treatment resulted in a significant extension of the global severe type SMA-like mice lifespan, which increased from 16 +/−10 days in vehicle-treated to 42+/−36 days in GKT137831-treated severe type SMA-like mice (Figure 5D).

Taken together, these data show that direct NOX4 inhibition in severe type SMA-like mice spinal cord significantly improves motor behavior, limits weight loss, and extends life expectancy of severe type SMA-like mice.

### 3.6. NOX4 inhibition acts synergistically with SMN exon-7 targeting ASOs to improve lifespan, weight gain, and motor behavior of severe type SMA-like mice

**Figure 6:**
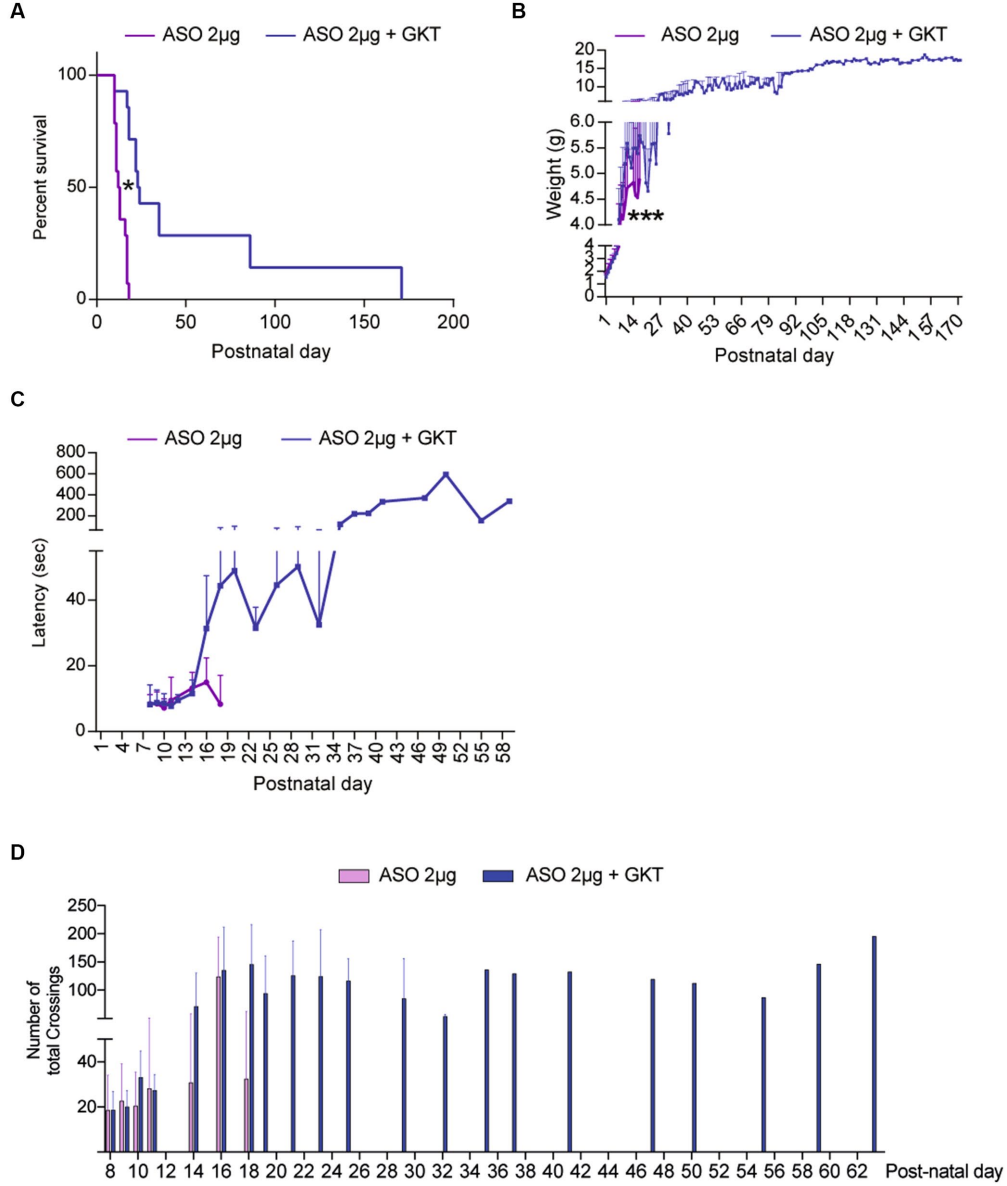
GKT137831 acts synergistically with ASOs targeting SMN exon-7 to improve SMA-like mouse lifespan. Lifespan **(A)** and body weight **(B)** Grip test **(C)** and number of total crossings in an open field **(D)** curves of severe type ASO-treated SMA-like mice co-treated with GKT137831 (pink) *per os* or vehicle (blue; *n* = 13; *p < 0.05 vs. SMA; error bars indicate SD). At p18, no ASO-treated mice was still alive while 9 ASO-treated SMA-like mice co-treated with GKT137831 were alive. At P26, 6 ASO-treated SMA-like mice co-treated with GKT137831 were alive. At P35, there was only one mouse alive that survive until p171.

Finally, we wondered whether the association of GKT137831 with the Nusinersen-like ASO 10–24 (ISIS-SMNRx), which efficiently promoted exon-7 inclusion in SMN mature transcripts in mice (Singh et al., 2009) could act synergistically *in vivo*. Hence, we treated at P1 a population of severe type SMA-like mice with a single dose of 2 μg ASOs, which constitutes the lowest dose of ASO affecting SMN expression (Rigo et al., 2014), and completed this treatment by repeated doses of 40 mg.g^−1^ of GKT137831 orally administered (*per os*) until mouse death. We first investigated the life span and body weight in ASO/GKT137831-treated severe type SMA-like mice in comparison to ASO-treated severe type SMA-like mice (Figure 6). Very interestingly, in the co-treated group, we recorded an increase in mouse median survival of 10 days (12.5 vs. 23 days in ASO- and ASO/GKT137831-treated SMA-like mice, respectively) and an increase in total survival (18+/−8 vs. 171+/−153 days in ASO- and ASO/GKT137831-treated severe type SMA-like mice, respectively; Figure 6A). When we looked at the body weight curves, we found that the ASO/GKT137831-treated severe type SMA-like mice maintained their body weight until death (Figure 6B). Finally, we investigated the potential impact of the co-treatment on mouse motor behavior. We found that the co-treatment increased the grip time of ASO-treated severe type SMA-like mice since the age of P17 (Figure 6C), and improved the mouse spontaneous activity as evaluated in an open field (Figure 6D).

Taken together, these data show that NOX4 inhibition acts synergistically with Nusinersen-like ASOs in the spinal cord of severe type SMA-like mice.

## 4. Discussion

In this study, we demonstrated that NOX4 is involved in SMA physiopathology and that its inhibition by GKT137831 induced a significant neuroprotection at the level of the spinal MNs, likely through the modulation of pro-survival signaling pathways. Moreover, we reported that the oral treatment with GKT137831 is likely to act synergistically with Nusinersen-like ASOs, even administered at minimal doses, to alleviate SMA symptoms *in vivo*, thus paving the way for using GKT137831 as a combinational therapy in SMA.

A major finding of this study is the deleterious role of NOX4 overexpression for SMA MNs. Among all enzymes involved in ROS generation, NADPH oxidases have for primary function to directly generate ROS to sustain specific cell needs, and not as byproducts of the reaction they catalyze (Coso et al., 2012). Accordingly, NOXs are expected to directly generate controlled levels of superoxide, or hydrogen peroxide in the specific case of NOX4, due to the rapid conversion of NOX4-generated superoxide to hydrogen peroxide (Shababi et al., 2012). Because NOX4 is constitutively active, its expression levels is directly associated to ROS production (Martyn et al., 2006; Nisimoto et al., 2010). Thus, although we did not studied the expression levels of other NOXs, the overexpression of NOX4 protein is likely to be responsible to generate ROS accumulation in the spinal cord of severe type SMA-like mice. Although the identity of the molecular mechanisms underlying NOX4 overexpression in SMA remains to be clarified, it should be noted that some signaling pathways controlling NOX4 expression have been previously shown deregulated in SMA. This is notably the case for the JAK-STAT3 pathway, that positively control NOX4 expression (Manea et al., 2010) which we (Biondi et al., 2015) and others (Ohuchi et al., 2019) have found constitutively overactivated in SMA spinal cord. Accordingly, re-equilibrating the JAK-STAT3 pathway in SMA spinal cord results in significant neuroprotection of spinal MNs (Biondi et al., 2015), as does NOX4 inhibition (this study).

**Figure 7:**
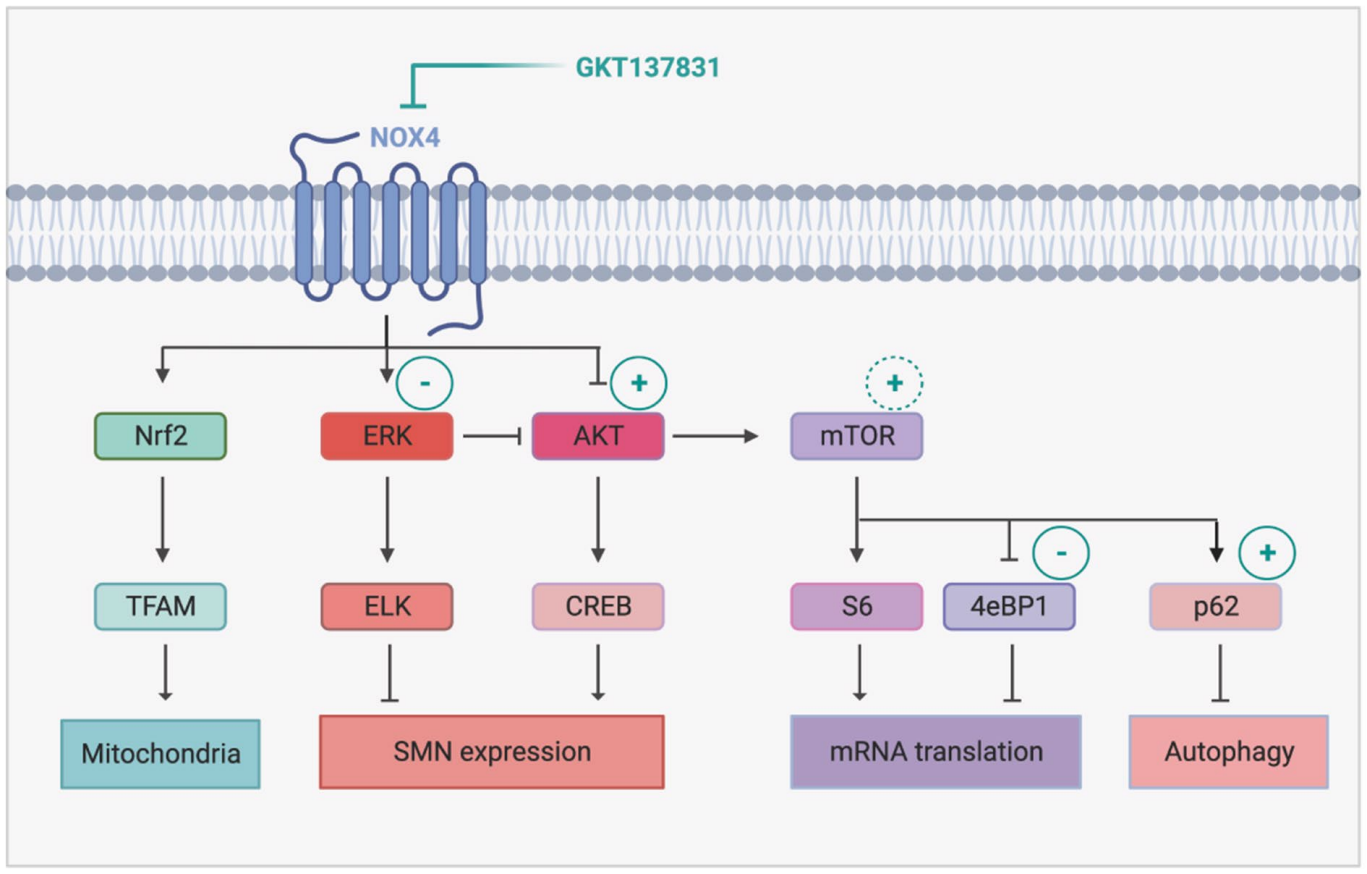
Proposed mechanism of NOX4 inhibition-induced modulation of intracellular signaling pathways in SMA. The sign + and − represents activation and inhibition of the signaling pathways following GKT137831 treatment, respectively. The inhibition of Nox4 decreases Erk activity and increases Akt activity. The activity of AKT promotes SMN increase through CREB activation. In addition, the activation of AKT leads to mTOR activation that enhances mRNA translation and autophagy. Created with BioRender.com.

Interestingly, NOX4 inhibition proved to alter the crosstalk between ERK and AKT in favor of AKT, which is quite unexpected considering the documented role of NOX4 in maintaining AKT activation through inhibition of tyrosine phosphatases (Cavaliere et al., 2013). NOX4 is also expected to activate ERK through the ROS it generates. In the present study, we found that NOX4 inhibition is sufficient to re-equilibrate ERK and AKT activation patterns, supporting the hypothesis of a NOX-dependent activation of ERK in SMA spinal cord (Figure 7). Conversely, activated ERK could activate NOX4 expression (Jeong et al., 2018), potentially resulting in an ERK-dependent maintenance of high levels of NOX4 expression. Accordingly, inhibition of NOX4 induced both the inhibition of ERK and the decrease in NOX4 protein expression levels (Figure 2E). Interestingly, the deletion of NOX2 in a mouse model of ALS, another MN neurodegenerative disease, was shown to induce efficient AKT activation in the mouse spinal cord associated with (i) a significant neuroprotection of the spinal MNs, (ii) a limitation of microgliosis and (iii) an extension of ALS mouse lifespan (Wu et al., 2006). Taken together with our results, these data suggest that, in the spinal cord, overexpressed NOXs negatively modulate the pro-survival AKT pathway, thus participating in MN death. Accordingly, their inhibition results in AKT induction, associated with spinal MN survival.

Moreover, we found that, downstream of AKT, NOX4 inhibition resulted in the activation of the transcription factor CREB and of the signaling platform mTOR (Figure 7). CREB is a well-established transcriptional activator of *SMN* genes (Branchu et al., 2013). Interestingly, the GKT137831-induced increase in the number of Gems in MN nuclei suggest in addition that the SMN protein produced is functional and active (Zhang et al., 2006). NOX4 inhibition resulted also in mTORC1 activation, as revealed by the induction of p4EBP1 phosphorylation. p4EBP1 is involved in initiation of cap-dependent translation (Jossé et al., 2016). Interestingly, impaired protein synthesis at the translational initiation step by inhibition of 4EBP1 phosphorylation has been previously reported in primary cultures of mouse SMA MNs (Thelen et al., 2020). Although in this work, Thelen and colleagues incriminated ROS generated by dis-functioning mitochondria, our results suggest that NOX4-generated ROS are also involved in these translation defects. Intriguingly, NOX4 inhibition was unlikely to increase S6 phosphorylation, another well described target of mTORC1 involved in translation control. However, in mature neurons, S6 phosphorylation is also dependent on ERK activity (Hartman et al., 2013). Thus, in SMA mouse spinal cord, S6 activation levels might be maintained in the same range, by ERK when NOX4 is overexpressed and by mTORC1 when NOX4 is inhibited by GKT137831. Interestingly, S6 is involved in NOX4 translation (Venado et al., 2016). The fact that NOX4 inhibition is unlikely to activate S6 might may prevent a positive retro-control loop on NOX4 (Shi et al., 2018) that would have attenuated the effects of its inhibition.

Most importantly, we report here that the combination of orally administered GKT137831 and a single minimal dose of Nusinersen-like ASOs induced synergistic beneficial effects on SMA symptoms *in vivo*. Transferred in patients treated with Nusinersen, a complementary oral GKT137831 treatment could lead to the reduction of intrathecal ASO injections in patients. Furthermore, the combinatory treatment resulted in the improvement of the global mouse health, including lifespan, body weight and motor and behavior phenotype. Indeed, the performance of SMA mouse group treated with the ASO and the GKT137831 level are close to the ones previously obtained for the WT mice (Biondi et al., 2010, 2015). These results suggest that the orally administered GKT137831 provides its beneficial effects on SMA-like mice by targeting damaged organs outside the CNS. Indeed, while there is no proof on the ability of GKT137831 to cross the brain–blood barrier, accumulating data sustain its role as a therapy in organs affected by SMA, such as lung, liver (Aoyama et al., 2012; Cao et al., 2017) and kidney (Jeong et al., 2018). Moreover, It has been shown that NOX4 expression is important for muscular physiology (Youm et al., 2019), suggesting that inhibition of NOX4 could restore the physiological condition and be beneficial for SMA muscles. Thus, the synergic effect of the combinatory treatment could come from the protection of MN by The ASO and of the peripheral tissue by GKT137831. There is no doubt that additional investigations need to be perform to identify which organs are likely to be improved by GKT137831 in SMA models, and whether NOX4 expression is also perturbated in human SMA patients. But the synergistic benefits we demonstrated here with the most largely used SMN-upregulating therapy to date, i.e., Nusinersen, strongly suggest that GKT137831/Setanaxib should be considered as a combinational approach to fight against SMA.

## Data availability statement

The original contributions presented in the study are included in the article/Supplementary material, further inquiries can be directed to the corresponding author.

## Ethics statement

The animal studies were approved by the Committee of the University of Paris Cité (APAFIS#31064–20,210,310,162,620). The studies were conducted in accordance with the local legislation and institutional requirements. Written informed consent was obtained from the owners for the participation of their animals in this study.

## Author contributions

MK designed and performed experiments, analyzed data, and wrote the manuscript. OB designed and analyzed the IF data. DS contributed to MN quantification and gems quantification. MK, ZC, EA, and CB contributed to mouse experiments. SB contributed to RT-qPCR experiments. GB designed and supervised the pre-clinical studies. FC and AE conceptualized the project. LW and FC designed the overall research, analyzed the data, and wrote the manuscript. All authors contributed to the article and approved the submitted version.

## Funding

ZC is the recipient of a Ph.D. fellowship from the Ministère de la recherche. This work was supported by Université Paris Cité and INSERM fundings.

## Conflict of interest

The authors declare that the research was conducted in the absence of any commercial or financial relationships that could be construed as a potential conflict of interest.

## Publisher’s note

All claims expressed in this article are solely those of the authors and do not necessarily represent those of their affiliated organizations, or those of the publisher, the editors and the reviewers. Any product that may be evaluated in this article, or claim that may be made by its manufacturer, is not guaranteed or endorsed by the publisher.

## Abbreviations

ASO: Antisense oligonucleotide
ALS: Amyotrophic Lateral Sclerosis
CNS: Central Nervous System
DHE: Dihydroethidium
MN: Motor Neuron
NMJ: Neuromuscular Junction
NOX4: NADPH oxidase 4
NRF2: Nuclear Factor Erythroid-derived 2 Related Factor 2
ROS: Reactive Oxygen Species
SMA: spinal muscular atrophy
SMN: Survival of Motor Neuron
TFAM: mitochondrial transcription factor A
