# Supplementary material for: NADPH oxidase 4 inhibition is a complementary therapeutic strategy for spinal muscular atrophy
Source: Front Cell Neurosci. 2023 Sep 14;17:1242828. doi: 10.3389/fncel.2023.1242828 (PMC10536974; doi:10.3389/fncel.2023.1242828)
Supplement: Supplementary file 1 [file Data_Sheet_1.pdf]

Supporting Information

Fig. S1

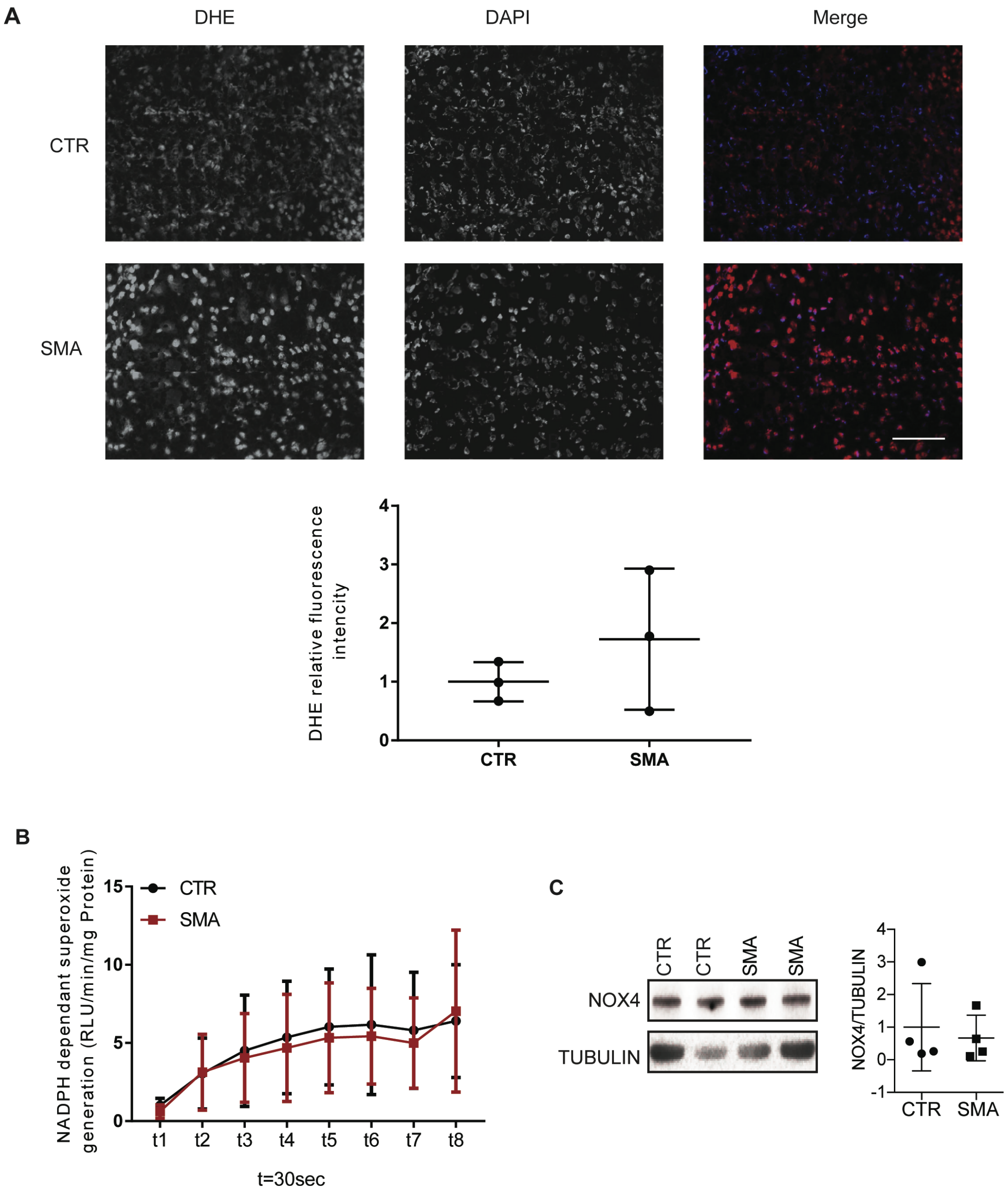

Figure S1. *ROS accumulation and NOX4 expression in the spinal cord of severe type SMA-like mice at P6*

A and **B.** Evaluation of superoxide accumulation by DHE staining (A) and of NADPH-dependent superoxide generation (B) in the spinal cord of severe type SMA-like mice compared to control P6 (n=4) (scale bar 50 µm). **C.** Western blot (left panel and quantification (right panel) of NOX4 and NOX1 protein expressions in the spinal cord of severe type SMA-like mice compared control mice at p6.

Fig. S2

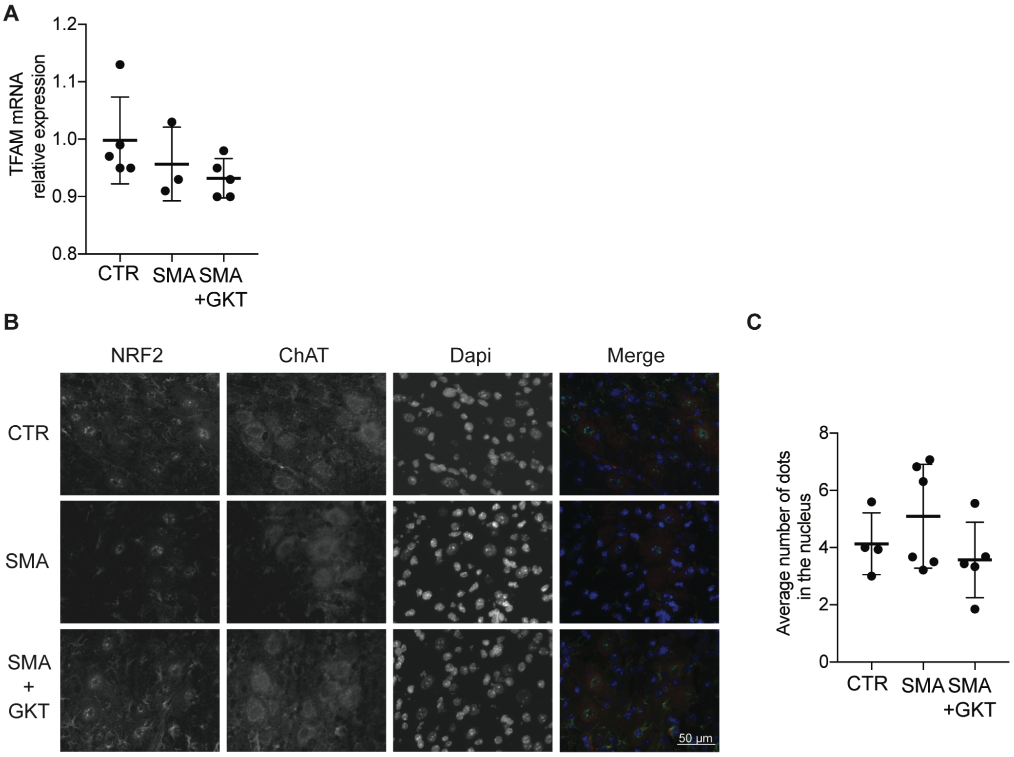

Figure S2. NOX4 inhibition does not affect the NRF2-TFAM pathway in the spinal cord of severe type SMA-like mice.

A. Analysis of the mRNA relative expression TFAM in the lumbar spinal cord of GKT137831- or vehicle-treated severe type SMA-like mice compared to control mice at P8 (n=3). TFAM expression was normalized by PPIA expression (Primer sequences are listed in the table S1) . **B.** **I**mmunodetection of NRF2 in ChAT- positive cells in the lumbar spinal cord of GKT137831- or vehicle-treated severe type SMA-like mice compared to control mice at P8 (H) (n=6). (Scale bar 50 µm). **C.** Quantification of Nrf2 dots number in the nucleus (*$p<0.05$; error bars indicate standard deviation).

	Forward	Reverse
**PPIA**	GGCAAATGCTGGACCAAAC	CATTCCTGGACCCAAAACG
**TFAM**	AACACCCAGATGCAAAACTTTCA	GACTTGGAGTTAGCTGCTCTTT

Table S1. Sequences of primers used for RT-qPCR

Supplementary Materiels

RNA extraction and RT-qPCR

Lumbar spinal cords were dissected and immediately frozen in liquid nitrogen. For RNA extraction, samples were mechanically homogenized in Trizol reagent (Invitrogen, Life Technologies) using metal beads and a TissueLyser apparatus (Qiagen). Then, extracted RNAs were ethanol precipitated.

Each RNA sample was first treated with RQ1 Dnase (Promega). Then, 1 µg of RNA was reverse transcribed with oligo(dT) (20 mer) using reverse transcriptase Improm II (Promega). Quantitative real time PCR was performed with standard protocols using Biorad CFX384 with SYBR Green ROX as a fluorescent detection and mouse primer at 100 nM (sequences are indicated in Table 1) in a final volume of 7 µL. The cDNAs for the real time PCR were used at 5 ng/µL. The calculated relative amount of mRNA was done respective to control samples and given as fold change after $2^{-}\Delta\Delta CT$ calculation [1].

1. Livak KJ, Schmittgen TD. Analysis of relative gene expression data using real-time quantitative PCR and the 2(-Delta Delta C(T)) Method. Methods. 2001;25:402–8.
